# Application of Coarse-Grained (CG) Models to Explore Conformational Pathway of Large-Scale Protein Machines

**DOI:** 10.3390/e24050620

**Published:** 2022-04-29

**Authors:** Danfeng Shi, Ke An, Honghui Zhang, Peiyi Xu, Chen Bai

**Affiliations:** 1 Warshel Institute for Computational Biology, School of Life and Health Sciences, The Chinese University of Hong Kong (Shenzhen), Shenzhen 518172, China; shidanfeng@cuhk.edu.cn (D.S.); kevinan@cuhk.edu.cn (K.A.); 221059040@link.cuhk.edu.cn (H.Z.); 117030076@link.cuhk.edu.cn (P.X.); 2 School of Chemistry and Materials Science, University of Science and Technology of China, Hefei 230026, China

**Keywords:** protein machines, coarse-grained model, conformational pathway

## Abstract

Protein machines are clusters of protein assemblies that function in order to control the transfer of matter and energy in cells. For a specific protein machine, its working mechanisms are not only determined by the static crystal structures, but also related to the conformational transition dynamics and the corresponding energy profiles. With the rapid development of crystallographic techniques, the spatial scale of resolved structures is reaching up to thousands of residues, and the concomitant conformational changes become more and more complicated, posing a great challenge for computational biology research. Previously, a coarse-grained (CG) model aiming at conformational free energy evaluation was developed and showed excellent ability to reproduce the energy profiles by accurate electrostatic interaction calculations. In this study, we extended the application of the CG model to a series of large-scale protein machine systems. The spike protein trimer of SARS-CoV-2, ATP citrate lyase (ACLY) tetramer, and P4-ATPases systems were carefully studied and discussed as examples. It is indicated that the CG model is effective to depict the energy profiles of the conformational pathway between two endpoint structures, especially for large-scale systems. Both the energy change and energy barrier between endpoint structures provide reasonable mechanism explanations for the associated biological processes, including the opening of receptor binding domain (RBD) of spike protein, the phospholipid transportation of P4-ATPase, and the loop translocation of ACLY. Taken together, the CG model provides a suitable alternative in mechanistic studies related to conformational change in large-scale protein machines.

## 1. Introduction

Protein machines are the basic units to perform biological functions in cells. By responding to biological stimulus such as substrate binding or nucleoside triphosphate hydrolysis, they can execute directional molecular movements or specific chemical reaction pathways, and they play an important role in the cycle of matter and energy in cells [1]. A large number of protein machines have been structurally resolved at the atomic level and functionally verified under physiological conditions, such as the natural “molecular motor” ATPase [2], the transmembrane protein GPCR that mediates cellular “communication” [3], and diverse enzymes that are responsible for the synthesis, decomposition, or transformation of substances [4]. Like the machines in macroscopic world, the steady-state or equilibrium conformations of these protein machines are often restricted to a small set of possibilities and occur in a specific order, which guarantees the highly organized activities of those machines [5]. In that way, the functions of protein machines are not only determined by their crystal structures at steady state, but also closely related to the conformational pathways connecting those states. Nowadays, the crystal structures at steady state are quite accessible through the application of cryo-electron microscopy, X-ray diffraction, or nuclear magnetic resonance [6]; however, the associated conformational pathways can hardly be characterized owing to the ephemerality and instability of intermediate states.

The technique of molecular dynamics simulation can reproduce conformational ensembles for a given molecule based on the application of empirical atomic force fields and the classical Newtonian dynamic equations, constituting an effective tool to analyze protein conformational changes. However, the exploration of conformational pathways for biological macromolecules are often limited by a series of factors such as the temporal or spatial scale of the involved biological processes, and the accuracy of empirical force fields [7]. For protein machine systems, subunits or domains that execute complete biological functions are often involved in thousands of residues, and the related conformational changes may consist of large-scale translocation or rotation of domains, which also makes the conformational exploration of the dynamic process of ultra-large-scale systems much more difficult.

The application of coarse-grained models is an effective approach for studying the conformational change of complex macromolecules and the associated energetics and dynamic features. In all CG models, a common purpose of reducing the freedom complexity of modeling systems was achieved by simplifying the amino acid side-chain or even the entire amino acid [8]. Depending on the different simplification strategies, a series of CG models such as Martini [9], AWSEM (associated memory, water mediated, structure, and energy model) [10], UNRES (united residue) [11], and CABS (C-alpha, beta, and sidechain) [12,13] models were developed depending on their representation of a single amino acid and force field design. By using one or two united atoms to represent the geometry of the main chain and side chains, the CG model developed by Warshel and coworkers initiated the development of intermediate resolution coarse-grained models, such as UNRES and CABS models, which were widely applied in the multiscale modeling for their favorable balance of speed and accuracy.

Here, we tried to expand the application of the CG model developed by Warshel and coworkers to a series of large-scale protein machines reported currently. The CG model was developed in the 1970s for solving the protein folding problem [14], and was continually refined into a well-accepted and powerful tool [15,16,17]. The most significant advantage of this method is its better treatment of electrostatics free energy than other CG models [15]. As almost all biological processes are controlled or regulated by electrostatic effects, the CG model accompanied by accurate electrostatic interaction evaluation can provide favorable energy profiles for the conformational pathways of protein systems. Subsequently, the method was also applied to the mechanism research of a series of complex systems, such as F1-ATPase [18,19,20,21], F0-ATPase [22,23,24], ribosome [25,26,27], and GPCRs [28,29,30]. Specifically, for membrane protein systems, the charges and parameters in the CG model have been carefully optimized and calibrated for the energetics of amino acid residues, peptide membrane insertions, and the evaluation of membrane protein stability [16]. The widespread adaptation of this model proves it to be an effective tool to depict the energy profile for the working process of different bio-systems.

In this study, we further applied CG models to conformational pathway analysis of large-scale biomolecular systems, including protein–protein interaction, enzymes, and transmembrane protein systems. A complete workflow comprising molecular modeling, acquisition of intermediate conformations, determination of electrostatic charge, and conformational free energy calculation was built and applied to describe the working processes of different protein machines from the perspective of energy profiles. Although these systems had different solvent environments, biological function, and conformational changes, the CG models can provide a reasonable trend of conformational free energy change. We hope that the energy analysis and conformational exploration of the CG model can be applied to more systems.

## 2. Methods

### 2.1. Coarse-Grained (CG) Model

The coarse-grained (CG) model applied in this work is based on the solvation of ionizable residues and emphasizes the electrostatic effects within proteins. In the CG model, the sidechain atoms are united into a simplified atom, while the atoms of the main chain are explicitly represented. The simplified atom is located at the mass center of side chains for polar and nonpolar residues, and at the center of the charged part for ionizable residues. The CG model is derived by fitting to the observed absolute stability, which means the folding free energy of proteins is expressed as:

(1) $$\begin{array}{cc} \Delta G_{fold} & =\Delta G_{main}+\Delta G_{side}+\Delta G_{main/side}\\ & =c_{1}\Delta G_{side}^{vdw}+c_{2}\Delta G_{solv}^{CG}+c_{3}\Delta G_{HB}^{CG}+\Delta G_{side}^{elec}+\Delta G_{side}^{polar}+\Delta G_{side}^{hyd}+\Delta G_{main/side}^{vdw} \end{array}$$

where $\Delta G_{side}^{vdw}$, $\Delta G_{solv}^{CG},\Delta G_{HB}^{CG},\Delta G_{side}^{elec},\Delta G_{side}^{polar}$,$\;\Delta G_{side}^{hyd},\;\mathrm{and}\;\Delta G_{main/side}^{vdw}$ represent the sidechain van der Waals interaction, the mainchain solvation energy, the mainchain hydrogen bond force, the sidechain electrostatic effect, polar, hydrophobic contribution, and the mainchain/sidechain van der Waals interaction, respectively. Scaling coefficients $c_{1}$, $c_{2}\;,\;$and $c_{3}$ have values of 0.10, 0.25, and 0.15, respectively, in this formula.

The mainchain interaction involves van der Waals interactions and electrostatic effects, and all bonding terms are calculated by ENZYMIX force field [31]. The key treatment for the CG model comes from the sidechain interaction. The sidechain electrostatic contribution can be expressed as:

(2) $$\Delta G_{side}^{elec}=-2.3RT\sum_{i}Q_{i}^{MC}\left(pKa_{i}^{i}-pKa_{i}^{w}\right)+\Delta G_{QQ}^{\left(f\right)}-\Delta G_{QQ}^{\left(uf\right)}+\Delta G_{Q}^{dev}$$

where $Q_{i}^{MC}$,$\;pKa_{i}^{i},pKa_{i}^{w},\Delta G_{QQ}^{\left(f\right)}$,$\;\Delta G_{QQ}^{\left(uf\right)},\;\mathrm{and}\;\Delta G_{Q}^{dev}$ stand for the Monte Carlo averaged charge, the *pKa* under protein environment, the *pKa* in water of the *i*th ionizable residue, the folded states interaction potential, the unfolded states interaction potential of ionized groups, and the term that describes the scaled-down effect of changing the protonation state of an ionizable residue upon unfolding, respectively. For the estimation of $pKa_{i}^{i}$, the Monte Carlo Proton Transfer (MCPT) method is utilized.

The Metropolis Monte Carlo (MC) approach is applied to determine the ionization states of protein residues under given pH and temperature [13,32]. Each MC step involves the attempt of proton transfer (PT) between a pair of ionizable residues or within an ionizable residue and bulk solvent. The transferring is repeated until the electrostatic interaction of the folded protein converges, then the ionization states of the protein residues are obtained to evaluate the CG free energy. During every MC move, the electrostatic free energy of a folded protein for the *m*th charge configuration $Q_{i}^{\left(m\right)}$ is given by:

(3) $$\Delta G_{elec}^{\left(m\right)}=-2.3RT\sum_{i}Q_{i}^{\left(m\right)}\left(pK_{a,i}^{i}-pH\right)+\Delta G_{QQ}^{\left(m\right)}$$

where $\Delta G_{QQ}^{\left(m\right)}$ represents the charge–charge interaction free energies in the *m*th charge configuration. When a lower value of electrostatic free energy is achieved or the Metropolis criteria are met, the charge configuration is accepted. The minimized $\Delta G_{elec}$ is used to evaluate the electrostatic contribution to the folding free energy using Equation (3).

The $pKa_{i}^{i}$ term is expressed as:

(4) $$pKa_{i}^{i}=pKa_{i}^{w}-\frac{sgn\left(Q_{i}^{ion}\right)}{2.3RT}\Delta G_{self,i}$$

where $sgn\left(Q_{i}^{ion}\right)\;$ refers to the charge sign function of the *i*th residue in its ionized state (which is +1 for HIS, ARG, LYS, and −1 for GLU, ASP). $\Delta G_{self,i}$ stands for the change in self-energy of an ionizable residue when it moves from water to the protein. $\Delta G_{self,i}$ is a key element for reliable evaluation of the electrostatic effect in the system. $\Delta G_{self,i}$ can be given by:

(5) $$\Delta G_{self,i}=\sum_{i}\left[U_{p}^{self}\left(N_{p}^{i}\right)+U_{np}^{self}\left(N_{np}^{i}\right)+U_{mem}^{self}\left(N_{mem}^{i}\right)\right]$$

where *U* is effective potential, and $U_{p}^{self},\;\;U_{np}^{self},\;\mathrm{and}\;U_{mem}^{self}$ are the self-energy contribution from non-polar residues, polar residues, and membrane grid points, respectively. $N_{p}^{i},\;N_{np}^{i},\;\mathrm{and}\;\;N_{mem}^{i}$ give the number of non-polar residues, polar residues, and membrane atoms surrounding the *i*th residue, respectively. The empirical function $U_{mem}^{self}$ is given by:

(6) $$U_{mem}^{self}=\{\begin{array}{c} U_{mem}^{self,ion,0}exp\left[-(\left(R_{min}-18\right)/12^{2}\right]\;\;\;\;\;\;\;R_{min}\leq 18\\ U_{mem}^{self,ion,0}\;\;\;\;\;\;\;\;\;\;\;\;\;\;\;\;\;\;\;\;\;\;\;\;\;\;\;\;\;\;\;\;\;\;\;\;\;\;\;\;\;\;\;\;\;\;\;\;\;\;\;\;R_{min}\leq 18 \end{array}$$

and

(7) $$U_{mem}^{self,ion,0}=\{\begin{array}{c} B_{mem}^{self,ion}exp\left[-0.2\left(N_{mem}-28\right)\right]\;\;\;\;\;\;\;0<N_{mem}\leq 28\\ B_{mem}^{self,ion}\;\;\;\;\;\;\;\;\;\;\;\;\;\;\;\;\;\;\;\;\;\;\;\;\;\;\;\;\;\;\;\;\;\;\;\;\;\;\;\;\;\;\;\;\;\;\;\;\;\;\;\;\;\;\;N_{mem}>28 \end{array}$$

where $R_{min}$ refers to the distance to the closest solvent molecule. The value of $B_{mem}^{self,ion}$ is 15 kcal/mol, which is based on the experimental data and computer simulations [33]. $N_{mem}$ is the number of neighboring membrane grid points. In this way, the self-energy penalty induced by a partial double counting in the center of the membrane can be avoided, so this CG model is suitable for studying complex protein systems such as transmembrane proteins [34].

### 2.2. Workflow Integrated with Coarse-Grained (CG) Model

Here, we present a molecular modeling workflow to explore the dynamic properties of protein machines in terms of their conformational pathways and free energy profiles. The complete workflow consists of four steps, namely, molecular modeling, acquisition of intermediate conformations, determination of electrostatic charge, and conformational free energy calculation.

Firstly, the initial endpoint assemblies connecting a specific biological process were built based on the crystal structures from the Protein Data Bank database [35]. The missing parts were repaired by Modeller software [36]. All the ligands were removed, and the proteins were trimmed to the same length. After that, targeted molecular simulation (TMD) [37] was conducted to construct the conformational pathways between different endpoint structures and sample a series of intermediate conformations representing the transition process. At each TMD step, the target structure was firstly aligned to the current structure, and then the root-mean-square (RMS) distance between the current structure and the target structure was computed. The force (*F_TMD_*) on each target atom is given by the equation:

(8) $$F_{TMD}=\frac{1}{2}\frac{k}{N}\left[RMS_{\left(t\right)}-RMS_{\left(0\right)}\right]$$

where the *k* represents the spring constant and N represents the number of targeted atoms. *RMS_(t)_* is the current RMS value. *RMS*_(0)_ is a reference value which evolves linearly from the initial RMSD to the final RMSD [37]. The initial structure was aligned to the target structure using the backbone heavy atoms, which were restrained to the initial positions by harmonic restraints with a force constant of 100.0 kcal/mol/Å^2^. The system was restrained throughout the simulation to prevent abnormal translation and rotation. When TMD from the initial state to the target state was completed, the initial and final states were interchanged, so we can obtain the intermediate structures of the whole transition process. Then, the accurate electrostatic charge was computed by the Monte Carlo Proton Transfer (MCPT) method. Finally, each structure was converted into coarse-grained (CG) representation and the CG free energy was calculated to obtain the conformational free energy profiles. For transmembrane proteins, membrane particles were added, and molecular dynamics relaxation was carried out to attain energy convergence. All simulations were completed by Molaris-XG software [31].

## 3. Application to Protein–Protein Interaction System

Protein–protein interaction (PPI) is the basic cooperation form wherein proteins perform their functions together. The formation of interfacial interactions is coupled with specific conformational states of each protein partner; therefore, the conformational change is an important regulatory mechanism for the binding and unbinding process of protein–protein interaction systems. Starting from the unbinding conformation, the protein will go through a series of intermediate conformations until it finally reaches another protein to form binding interactions. The energic profile of a protein’s conformational change process can explain detailed mechanisms for the existence of a PPI relationship with its partner.

**Figure 1:**
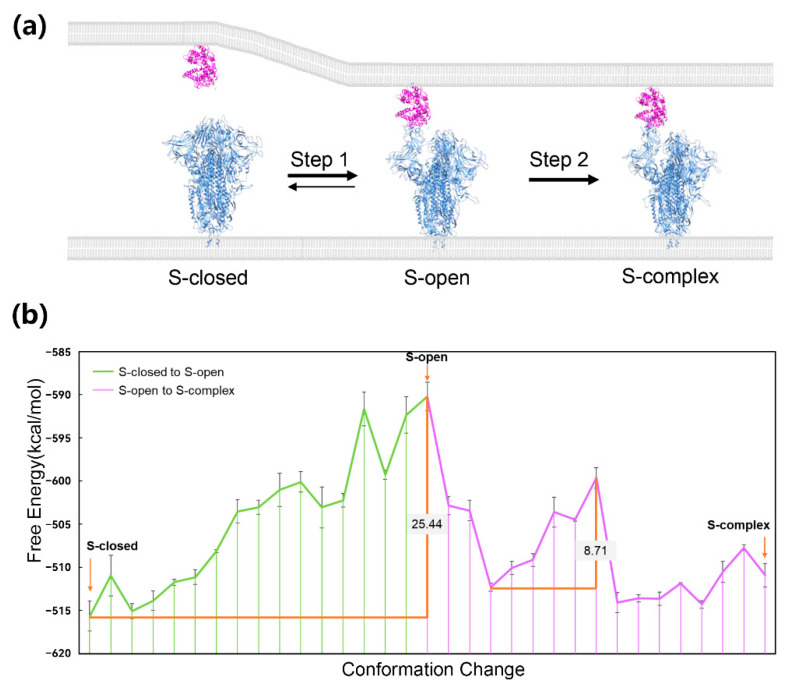
The conformational change process of the spike protein and the energic profile of the process. (**a**) Three conformations of the spike protein of SARS-CoV-2 (blue) and the position of the ACE2 receptor (mega); the conformational changes are presented as arrows (**b**). The energic profile of the two steps and the energy barriers are presented as orange lines.

The spike protein of SARS-CoV-2 is a typical molecular machine that depends on conformational changes to form protein–protein interaction. It locates on the surface of the viral envelope and mediates the viral entry into cells by interacting with the ACE2 receptor of human cells through its receptor binding domain (RBD) [38,39]. A complete spike protein is a homotrimer with a large scale of more than 3000 residues. There are three distinct conformational states of spike trimers in nature: S-closed, S-open, and S-complex (Figure 1a) [40]. S-closed is a tightly closed state in which the receptor binding motif (RBM) is masked, while S-open is a fusion-prone state with one RBD erecting to expose the RBM. Under the ACE2-free condition, most spike trimers are in the S-closed state and a minor population of them are in the S-open state, forming a dynamic balance (Figure 1a). During ACE2 approaching, the conformational landscape shifts toward the S-open state, and finally, most trimers reach the state in which they can tightly bind to ACE2 (Figure 1a). The transition process reflects the free energy difference among the three conformational states of spike trimers. Due to the too many computational resources needed by such a large-scale system, the energic profile of the process has never been obtained.

Here, we constructed CG models of the spike protein to reduce the resource demand for molecular modeling. The structural models of three conformations of the spike trimer were built by the Cryo-EM structures (PDB ID: 7DF3, 7DK3, and 7DF4) resolved by Xu et al. [40]. Then, the molecular modeling workflow integrated with the CG model was performed to obtain the energic profile of the process. Our results give an adequate explanation about behavior characteristics of spike proteins. Regarding the relative free energy of the three experimental conformations, S-closed has the lowest free energy, and the free energy of S-complex is the second lowest, while that of S-open is the highest (Figure 1b, Table 1). This gives an explanation that most spike trimers are in the S-closed state because of the energy advantages under ACE2-free conditions. The physiological significance of the S-closed state is hiding RBD, which is recognized by antibodies to achieve immune evasion, and therefore, the viruses become more adaptable [40]. During the transition process, the conformational free energy keeps rising until reaching the peak, which corresponds to the S-open conformation, and then turns into a downward trend (Figure 1b). The energy barrier of the spike trimer’s conformational transition is 25.44 kcal/mol (Figure 1b). Other computational studies also obtained an energy barrier varying from 7 to 20 kcal/mol of the process [41,42,43]. The high energy barrier is consistent with the conclusion that SARS-CoV-2 spike protein has higher energy barriers between active and inactive states as compared with the SARS-CoV-1 spike protein [44]. Lu et al. verified the high barrier between S-closed and S-open states by performing single-molecule fluorescence (Förster) resonance energy transfer experiments [45]. The evolutionary significance of the high energy barrier is to maintain the S-closed state of most trimers to hide surface antigens. Our calculation here only reflects the barrier of the conformational change in spike trimers. In reality, the energy barrier of spike protein’s activation may be different due to the role of other ligands, such as glycans [41,46]. This suggests that the S-open state is the energy barrier site for the whole conformational change process. The approaching of ACE2 may shift the balance of S-closed and S-open states [40] by reducing the energy barrier. We decomposed the energic terms of free energy of S-closed and S-open, and found that the main contribution is that S-open’s hydrophobic energy is much weaker than that of S-closed (Table 1). This is reasonable because RBD’s erecting exposes RBM as well as lots of hydrophobic residues in the solvent. We previously reported the important role of spike/ACE2 interaction in variants of SARS-CoV-2 [47,48]. The interaction between ACE2 and S-open may compensate the hydrophobic energy and contribute more electrostatic energy than the interaction between ACE2 and S-closed because of the glycans covering RBD [49,50]. The free energy of the S-complex conformation is lower than S-open but is still hard to reach because there is another energy barrier in the conversion process from S-open to S-complex (Figure 1b). As the hydrophobic energy of S-complex is stronger than S-open (Table 1), when ACE2 binding, the S-complex state could have a significantly reduced free energy and become the most stable conformation.

## 4. Application to Enzyme System

As a special class of protein machines, enzymes reduce the energy barriers of chemical reactions with the help of key residues or cofactors under the protein environment. Upon substrate binding, an enzyme may undergo a wide range of conformational changes, but only some specific intermediate states are important and meaningful for their stabilities on kinetic and thermodynamic properties [51]. It is hypothesized that the catalytic abilities of enzymes are dependent on the stabilities of transition-state structures of the substrate compared to that of the ground state [52]. Revealing the atomic details and energy change along the conformational transition process could provide preliminary mechanistic explanation of the catalytic process. In this case, we take ATP citrate lyase (ACLY) as an example and describe its key conformational pathway the during substrate-binding process.

**Figure 2:**
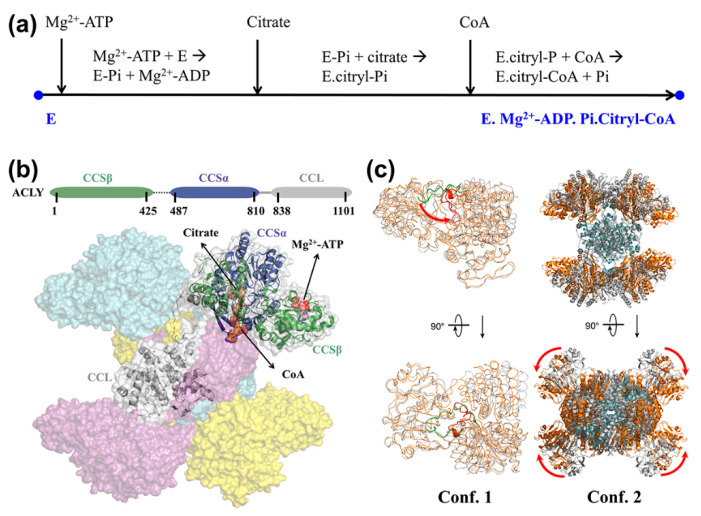
The introduction of structure and function of ACLY tetramer. (**a**) The complete substrate-binding process and related chemical reactions for ACLY. The blue points at both terminals represent the apo structure and ligand-binding structure for ACLY. (**b**) The monomer sequence and tetramer structures of ACLY. The binding sites of Mg^2+^-ATP, citrate, and CoA are labeled with black arrows. (**c**) Two main conformational changes during the substrate-binding process. Conf. 1 represents the translocation of His760-located loop; conf. 2 represents the conformational rotation of the CCS domain relative to CCL domain.

ATP-citrate lyase (ACLY) is a representative central metabolic enzyme linking carbohydrate and lipid metabolism [53]. It catalyzes coenzyme A (CoA) to acetyl coenzyme A (Ac-CoA) with the participation of Mg^2+^-ATP and citrate. The overall reaction is as follows: Mg^2+^-ATP + Citrate + CoA → Mg^2+^-ADP + Pi + Ac-CoA + oxaloacetate. Its substrate-binding process is accomplished with the successive binding of Mg^2+^-ATP, Citrate, CoA, and the related chemical reactions, as shown in Figure 2a. The latest crystallographic studies have revealed that the basic function unit of ACLY is a homo-tetrameric complex with D2 symmetry (Figure 2b), comprising four CCL domains that closely assemble at the center of ACLY tetramer and four CCS domains that freely stretch outside [54,55,56]. Two significant conformational changes in ACLY tetramers were observed from the crystal structures at the endpoints of the substrate-binding process (Figure 2c): (1) the His760 located loop transferred from the Mg^2+^-ATP site to the citrate site after reaction (i), and (2) the CCS domain on each monomer underwent significant conformational changes compared to the CCL domain. Owing to the structural lack of His760-located loop in both apo and substrate-binding crystal structures, it is unclear whether these two conformational changes occur in cascade or in coupled ways. Therefore, we tried to construct a detailed free energy profile of conformational change and provide a clear mechanistic explanation.

Here, the ACLY tetramers along the substrate-binding process were built based on the D2-symmetrical Cryo-EM structures solved by Wei et al. [56]. The two captured conformations E and E’ (PDB ID: 6POF, 6UUW) represent the start point and end point conformations of the substrate-binding process, respectively. It is indicated that the long linker region between CSSβ and CSSα (sequence 426-486) did not affect the catalytic function of ACLY tetramer [54]. Therefore, all the protein monomers were trimmed to the same length (sequence 2-425, 487-1099). The missing loop of conformation E (sequence 751-766) and conformation E’ (sequence 140-148) were completed from the corresponding section of another ACLY tetramer (PDB ID: 6QFB). Then, the molecular modeling workflow integrated with the CG model was performed to obtain the energic profile of the process.

**Figure 3:**
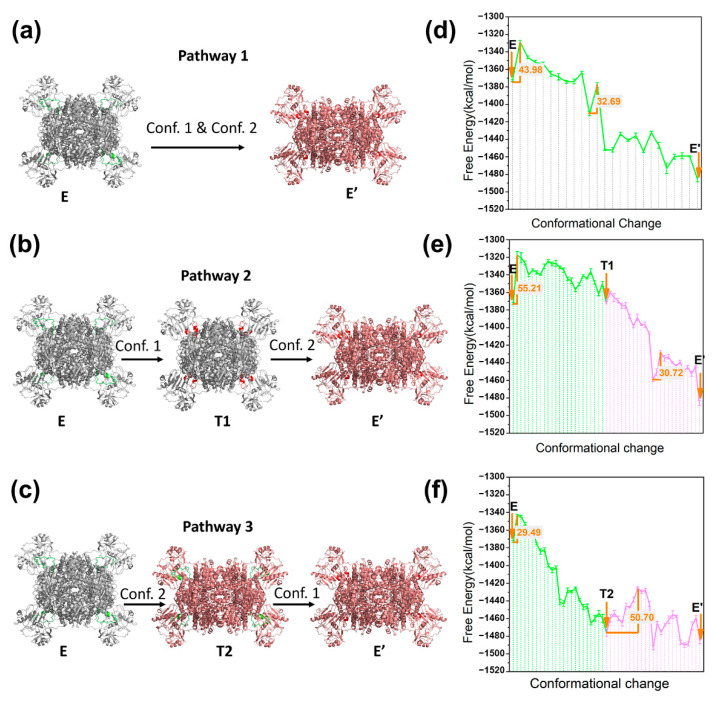
Three possible conformational pathways for ACLY during the substrate-binding process and the corresponding energy profile. E, E’, T1, and T2 represent starting state, ending state, and intermediate states 1 and 2, respectively. (**a**,**d**) Pathway 1 (E→E’); (**b**,**e**) pathway 2 (E→T1→E’); (**c**,**f**) pathway 3 (E→T2→E’). The main energy barriers are calculated and labeled in orange.

As shown in Figure 3a–c, according to the occurrence order of two conformational changes (conf. 1 and conf. 2), a coupling pathway (pathway 1) and two cascade pathways (pathway 2, pathway 3) were constructed, in which E, E’, T1, and T2 represent the starting state, ending state, and intermediate states 1 and 2, respectively. The calculated conformational free energies for state E, E’, T1, and T2 are −1372.78 kcal/mol, −1485.72 kcal/mol, −1371.95 kcal/mol, and −1476.02 kcal/mol, respectively (Table 1). The energy difference between E and E’ was −112.94 kcal/mol, indicating that the conformational change in ACLY showed a stable trend during the substrate-binding process. The energy difference between E and T1 was slight, while the energy difference between E and T2 was approximately 100 kcal/mol, indicating that conf. 2 but not conf. 1 causes a major energy change during the process. It can be seen that the energy difference between E and E’ was mainly affected by the electrostatic energy term (about 45 kcal/mol) and hydrophobic energy term (about 60 kcal/mol), as shown in Table 1. According to the conformational analysis by Wei et al. [56], E’ (the substrate-binding pose) showed a more compact position between monomers relative to E (the apo pose), which partly suggests the high stability of the conformation E’. The energy profiles of three pathways were further analyzed, as shown in Figure 3d–f. In the coupled situation, two important energy barrier changes are located in the initial and middle stages of the conformational change of pathway 1 with values of 43.98 kcal/mol and 32.69 kcal/mol, respectively. As for pathway 2, the energy barrier of E→T1 is 55.21 kcal/mol, while the energy barrier of T1→E’ is 30.72 kcal/mol. In pathway 3, the energy barrier of E→T2 stage is 29.49 kcal/mol, while the energy barrier of T2→E’ is 50.70 kcal/mol. It can be seen that conf. 1 and conf. 2 correspond to two important energy barrier changes in the free energy profile. At the E→T1 of pathway 2 and the T2→E’ of pathway 3, the energy barrier values are both higher than 50 kcal/mol, indicating that conf. 1 corresponds to the key energy barrier in the overall substrate-binding process. In the cascade of chemical reactions of the ACLY catalytic cycle, the activation effect of Mg^2+^-ATP at the starting point is the driving force of the conformational shift of His760-located loop [57]. The above results provide a mechanistic explanation for the necessity of Mg^2+^-ATP activation in the catalytic cycle of ACLY. A comparison between the energy barriers of three pathways shows that the coupling of conf. 1 and conf. 2 in pathway 1 could effectively reduce the highest energy barriers of conf. 1 (11.23 kcal/mol lower than pathway 2, and 6.72 kcal/mol lower than pathway 3), which preliminarily showed the synergy between different domains during the conformational change process.

## 5. Application to Transmembrane Protein System

Transmembrane proteins are integral to many biological processes such as membrane trafficking, enzymatic activity, and cell–cell recognition, to name a few [58]. The function of transmembrane proteins is achieved by the conformational changes under the effect of membrane constituents [59]. It is a challenge to investigate the precise conformational change details of protein machines in the presence of membranes. P4-ATPase is an important transmembrane protein that acts as an essential transporter to flip specific lipids from the extracellular leaflet to the inner leaflet of cell membranes in eukaryote cells [60]. This transporting activity regulates nearly all biological process such as myotube formation, apoptosis, immune response, and sperm capacitation [61,62,63,64,65,66,67]. Understanding how phospholipid and proteins within bilayers operate and interact represents an important question in health and pathophysiology. Massive endeavors have been undertaken to glean structural and mechanistic insights into P4-ATPase over the decades [68,69,70,71]. With the technical development of Cryo-EM and X-ray crystallography, we know that P4-ATPases are made up of α protein subunit and β cell division cycle (CDC) 50 subunit. The α subunit consists of the A domain (actuator domain), N domain (nucleotide binding domain), P domain (phosphorylation domain), and 10 TMs (transmembrane helix). Without the CDC50 expression, P4-ATPase cannot induce catalytic activity [72]. Further mutagenesis and assay experiments provide insights into the key amino acids that are indispensable for the lipid recognition and for the translocation pathway [68,73]. However, the energy basis of the catalytic process by P4-ATPase is still not well understood.

**Figure 4:**
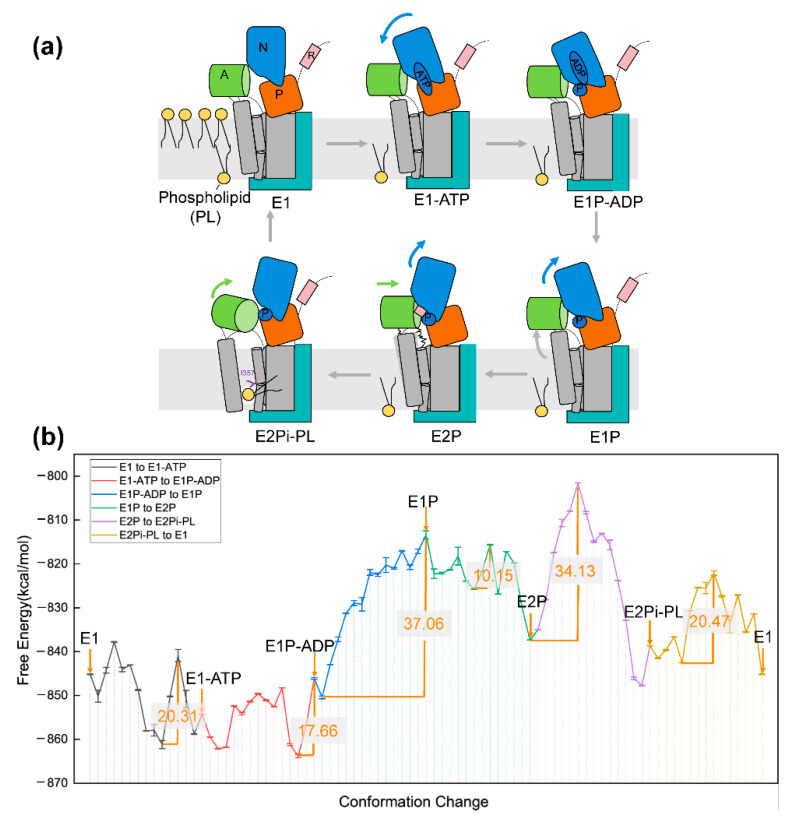
The schematic diagram for conformational change of P4-ATPase and the energic profile during the phospholipid translocation cycle. (**a**) The phospholipid translocation cycle of P4-ATPase. (**b**) The free energies for different conformational transition structures. The energy barriers are shown in orange.

Here, the initial models were constructed based on the Cryo-EM structures solved by Hiraizumi and coworkers [71]. The six captured intermediates E1, E1-ATP, E1P-ADP, E1P, E2P, and E2Pi-PL (PDB ID: 6K7G-6K7M) reflect the whole reaction cycle of lipid transfer (Figure 4a). The membrane was added by Molaris-XG software [31]. The membrane model was selected in which the hydrophobic contributions are scaled down by a factor of ∼3.6 and does not consider the polar contribution of sidechains [16]. The free energy for the six endpoint states of P4-ATPase system is displayed in Table 1. The highest energy of E1P state (−813.39 kcal/mol) suggests its instability in the observed conformation, which is consistent with the finding that E1P is a transient phosphorylated state [71]. E1P connects the phosphoryl transfer intermediate (E1P-ADP) and phosphoenzyme ground state (E2P), during which the TM1 and TM2 segments in the region proximal to the A domain clearly shift. Moreover, the distance between the N and P domain elongate when the N domain forces out in the E1P state [71]. Such structural change may induce the lower electrostatic energy term (Table 1). The obviously low value of E_Form2MC_ (−27.82 kcal/mol) in the E1P state conforms to the assumption that the electrostatic term usually contributes the most from the different interaction items in biophysical systems [21].

We also analyzed the whole energy transition for the transport cycle along the lipid flipping reaction, as shown in Figure 4b. The energy barrier (37.06 kcal/mol) for the transition from E1P-ADP to E1P surpass the values of the resting transitions. This is consistent with the fact that the interaction network in the E1P-ADP state reconnects within a short time during the dissociation of ADP, and energy generated from ATP hydrolysis is needed to compensate. For other ATPase members such as F1-ATPase and Adenosine triphosphate (ATP)-binding cassette (ABC) transporter, the energy barrier for conformation change varies differently from 10 to 32.1 kcal/mol [23,74,75]. The relatively high barrier of P4-ATPase may be due to the relatively large molecular conformation and functional specificity of phospholipid transporters [76]. The second important energy barrier (34.13 kcal/mol) occurs at the transition from E2P to E2Pi-PL, indicating that proteins need to overcome potential resistance generated by the occlusion of lipid head groups. The energy barrier is obtained at the early stage of the transition, representing that phospholipids on the exoplasmic side of the membrane invade the membrane at the start of the transition from E2P to E2Pi-PL. This is consistent with the cellular observations of Nakanishi and coworkers [77]. With our coarse-grained simulation methods, we can achieve relative reasonable results and give a quantitative explanation for the phospholipid transport progress.

## 6. Conclusions and Summary

Here, we reported the application of the CG model on several large-scale protein machine systems. It is important to mention that our results still follow the philosophy of capturing more relevant physical features and focusing less on minute details [78]. It is not a perfect strategy, but in many cases, it gives the best option for capturing the functions of complex biological systems. The spike protein of SARS-CoV-2 is an important target for antiviral drugs because of its critical role in infection [79]. We modeled its three conformations by using the CG model and calculated the whole energetic profile of conformational change. The results explain the energetic basis of the mechanism of spike protein conformational change. Furthermore, ACLY is a homo-tetrameric structure with D2 symmetry; hence, its conformational changes are accompanied by more complex monomer motions and coupling relationships. We detailed here the coupling process of conformational changes of the ACLY enzyme during its catalytic cycle. Finally, we studied the catalytic mechanism of a membrane protein system, P4-ATPase, which is responsible for lipid transport across membranes. The difficulty in modeling this system is that it embedded in a membrane environment and has six intermediate conformations. The phospholipid transport function is accomplished in a cycle of successive transitions of these six conformations. Thanks to the optimization of membrane proteins of the CG models, we described the energetic profile of the conformational change cycle and gave a quantitative explanation for the phospholipid transport progress. These applications demonstrate valid examples of the applicability of the CG model in large-scale protein machines.

With the development and application of cryo-electron microscopy, more and more intermediate conformation states of protein machine systems will be resolved, and the CG model will be more widely applied in this context. The most important application of the CG model is in studying the coupling of a protein’s conformational changes with specific reactions. Most protein machines cannot function solely by conformational transitions and need to be coupled with interactions with other biomolecules. For instance, the spike proteins must interact with the ACE2 receptor to invade cells; the catalytic reaction of ACLY involves the substrate binding of CoA, Mg^2+^, and ATP to carry out chemical reactions; and the lipids need to bind to P4-ATPase to be transported. There is no doubt that the participation of these biomolecules may promote or depend on the conformational changes of the protein machines. It is important to understand the coupling relationship between conformational change and biological events and then gain a deeper understanding of protein functions. Achieving this goal requires more efficient modeling of the free energy landscape of biological process, which must extend the application of the CG model by combining it with other computational methods—for example, using the empirical valence bond (EVB) method [80] to compute the energy profile of chemical reactions in protein, using the protein dipoles Langevin dipoles (PDLD) method [81] to calculate the binding free energy between a ligand and a protein, etc. Altogether, based on its advantage of conformational free energy evaluation, the CG model is set to play an increasingly important role in various computational biology studies.

## Figures and Tables

**Table 1:** Conformational free energy terms of experimental conformations of spike protein, ACLY tetramer, and P4-ATPase systems. The unit of energy is kcal/mol.

	E_Form2MC_^1^	E_Scaled size_^2^	E_Hydro_^3^	E_VDW_^4^	E_-DG UF_^5^	E_POLAR_^6^	E_total_^7^	STD ^8^
**Spike protein**								
S-closed	−421.71	578.35	−683.64	−49.01	4.38	−44.01	−615.65	1.74
S-open	−424.53	578.35	−658.64	−46.55	4.38	−43.21	−590.20	1.66
S-complex	−426.12	578.35	−677.86	−46.51	4.38	−43.13	−610.89	1.37
**ACLY tetramer**								
E	−990.30	706.79	−1014.88	−57.54	1.07	−17.92	−1372.78	1.01
E’	−1035.79	706.79	−1073.75	−63.33	1.07	−20.71	−1485.72	2.93
T1	−983.19	706.79	−1021.02	−57.24	1.07	−18.36	−1371.95	1.37
T2	−1038.84	706.79	−1061.91	−63.20	1.07	−19.93	−1476.02	4.30
**P4-ATPase**								
E1	−57.31	254.49	−960.92	−20.74	0.96	−61.64	−845.16	0.10
E1-ATP	−77.34	254.49	−945.38	−21.01	0.96	−65.96	−854.24	0.04
E1P-ADP	−61.12	254.49	−959.66	−21.05	0.96	−59.73	−846.11	0.21
E1P	−27.82	254.49	−952.45	−20.94	0.96	−67.63	−813.39	0.96
E2P	−50.63	254.49	−946.14	−23.20	0.96	−70.50	−835.02	0.02
E2Pi-PL	−40.35	254.49	−967.79	−21.09	0.96	−64.88	−838.66	0.04

^1^ Electrostatic energy term obtained using whole residue charges (0 or ±1), which minimize electrostatic energy in the MCPT method. ^2^ Empirical term that takes into account the effect of protein size on folding free energy. ^3^ Scaled hydrophobic energy term. ^4^ Scaled van der Waals energy term. ^5^ Negative of a scaled charge–charge energy estimate of an unfolded protein. ^6^ Polar energy contribution term. ^7^ The sum of E_Form2MC_, E_Scaled size_, E_Hydro_, E_VDW_, E_-DG UF_, and E_POLAR_. ^8^ The standard deviation.

## Data Availability

Data sharing is not applicable.

## References

[1] Piccolino M. (2000). Biological machines: From mills to molecules. Nat. Rev. Mol. Cell Biol..

[2] Iino R., Kinbara K., Bryant Z. (2020). Introduction: Molecular motors. Chem. Rev..

[3] Hauser A.S., Kooistra A.J., Munk C., Heydenreich F.M., Veprintsev D.B., Bouvier M., Babu M.M., Gloriam D.E. (2021). GPCR activation mechanisms across classes and macro/microscales. Nat. Struct. Mol. Biol..

[4] Hauer B. (2020). Embracing nature’s catalysts: A viewpoint on the future of biocatalysis. ACS Catal..

[5] Micheletti C., Banavar J.R., Maritan A. (2001). Conformations of proteins in equilibrium. Phys. Rev. Lett..

[6] Helliwell J.R. (2021). Combining X-rays, neutrons and electrons, and NMR, for precision and accuracy in structure–function studies. Acta Crystallogr. A.

[7] Hollingsworth S.A., Dror R.O. (2018). Molecular dynamics simulation for all. Neuron.

[8] Kmiecik S., Gront D., Kolinski M., Wieteska L., Dawid A.E., Kolinski A. (2016). Coarse-grained protein models and their applications. Chem. Rev..

[9] De Jong D.H., Singh G., Bennett W.F.D., Arnarez C., Wassenaar T.A., Schafer L.V., Periole X., Tieleman D.P., Marrink S.J. (2013). Improved Parameters for the Martini Coarse-Grained Protein Force Field. J. Chem. Theory Comput..

[10] Davtyan A., Schafer N.P., Zheng W., Clementi C., Wolynes P.G., Papoian G.A. (2012). AWSEM-MD: Protein structure prediction using coarse-grained physical potentials and bioinformatically based local structure biasing. J. Phys. Chem. B.

[11] Liwo A., Baranowski M., Czaplewski C., Golas E., He Y., Jagiela D., Krupa P., Maciejczyk M., Makowski M., Mozolewska M.A. (2014). A unified coarse-grained model of biological macromolecules based on mean-field multipole-multipole interactions. J. Mol. Model..

[12] Kolinski A. (2004). Protein modeling and structure prediction with a reduced representation. Acta Biochim. Polym..

[13] Messer B.M., Roca M., Chu Z.T., Vicatos S., Kilshtain A.V., Warshel A. (2010). Multiscale simulations of protein landscapes: Using coarse-grained models as reference potentials to full explicit models. Proteins.

[14] Levitt M., Warshel A. (1975). Computer simulation of protein folding. Nature.

[15] Vicatos S., Rychkova A., Mukherjee S., Warshel A. (2014). An Effective Coarse-Grained Model for Biological Simulations: Recent Refinements and Validations. Proteins.

[16] Vorobyov I., Kim I., Chu Z.T., Warshel A. (2016). Refining the treatment of membrane proteins by coarse-grained models. Proteins.

[17] Schopf P., Warshel A. (2014). Validating Computer Simulations of Enantioselective Catalysis; Reproducing the Large Steric and Entropic Contributions in Candida Antarctica Lipase B. Proteins.

[18] Mukherjee S., Bora R.P., Warshel A. (2015). Torque, chemistry and efficiency in molecular motors: A study of the rotary-chemical coupling in F1-ATPase. Q. Rev. Biophys..

[19] Mukherjee S., Warshel A. (2015). Brønsted slopes based on single-molecule imaging data help to unveil the chemically coupled rotation in F1-ATPase. Proc. Natl. Acad. Sci. USA.

[20] Mukherjee S., Warshel A. (2015). Dissecting the role of the γ-subunit in the rotary-chemical coupling and torque generation of F1-ATPase. Proc. Natl. Acad. Sci. USA.

[21] Mukherjee S., Warshel A. (2011). Electrostatic Origin of the Mechanochemical Rotary Mechanism and the Catalytic Dwell of F1-Atpase. Proc. Natl. Acad. Sci. USA.

[22] Bai C., Warshel A. (2019). Revisiting the protonmotive vectorial motion of F0-ATPase. Proc. Natl. Acad. Sci. USA.

[23] Bai C., Asadi M., Warshel A. (2020). The catalytic dwell in ATPases is not crucial for movement against applied torque. Nat. Chem..

[24] Mukherjee S., Warshe A. (2012). Realistic Simulations of the Coupling between the Protomotive Force and the Mechanical Rotation of the F-0-Atpase. Proc. Natl. Acad. Sci. USA.

[25] Rychkova A., Mukherjee S., Bora R.P., Warshel A. (2013). Simulating the Pulling of Stalled Elongated Peptide from the Ribosome by the Translocon. Proc. Natl. Acad. Sci. USA.

[26] Adamczyk A.J., Warshel A. (2011). Converting Structural Information into an Allosteric-Energy-Based Picture for Elongation Factor Tu Activation by the Ribosome. Proc. Natl. Acad. Sci. USA.

[27] Sharma P.K., Xiang Y., Kato M., Warshel A. (2005). What Are the Roles of Substrate-Assisted Catalysis and Proximity Effects in Peptide Bond Formation by the Ribosome?. Biochemistry.

[28] Alhadeff R., Warshel A. (2020). A free-energy landscape for the glucagon-like peptide 1 receptor GLP1R. Proteins.

[29] Bai C., Wang J., Mondal D., Du Y., Ye R.D., Warshel A. (2021). Exploring the activation process of the β2AR-Gs complex. J. Am. Chem. Soc..

[30] Alhadeff R., Vorobyov I., Yoon H.W., Warshel A. (2018). Exploring the free-energy landscape of GPCR activation. Proc. Natl. Acad. Sci. USA.

[31] Lee F.S., Chu Z.T., Warshel A. (1993). Microscopic and semimicroscopic calculations of electrostatic energies in proteins by the POLARIS and ENZYMIX programs. J. Comput. Chem..

[32] Beroza P., Fredkin D.R., Okamura M.Y., Feher G. (1991). Protonation of interacting residues in a protein by a Monte Carlo method: Application to lysozyme and the photosynthetic reaction center of Rhodobacter sphaeroides. Proc. Natl. Acad. Sci. USA.

[33] Dryga A., Chakrabarty S., Vicatos S., Warshel A. (2012). Coarse grained model for exploring voltage dependent ion channels. Biochim. Biophys. Acta.

[34] Fan Z.Z., Hwang J.K., Warshel A. (1999). Using simplified protein representation as a reference potential for all-atom calculations of folding free energy. Theor. Chem. Acc..

[35] Burley S.K., Bhikadiya C., Bi C., Bittrich S., Chen L., Crichlow G.V., Christie C.H., Dalenberg K., Di Costanzo L., Duarte J.M. (2021). RCSB Protein Data Bank: Powerful new tools for exploring 3D structures of biological macromolecules for basic and applied research and education in fundamental biology, biomedicine, biotechnology, bioengineering and energy sciences. Nucleic Acids Res..

[36] Webb B., Sali A. (2016). Comparative Protein Structure Modeling Using MODELLER. Curr. Protoc. Bioinform..

[37] Schlitter J., Engels M., Krüger P. (1994). Targeted molecular dynamics: A new approach for searching pathways of conformational transitions. J. Mol. Graph..

[38] Rabaan A.A., Al-Ahmed S.H., Haque S., Sah R., Tiwari R., Malik Y.S., Dhama K., Yatoo M.I., Bonilla-Aldana D.K., Rodriguez-Morales A.J. (2020). SARS-CoV-2, SARS-CoV, and MERS-COV: A comparative overview. Infez. Med..

[39] Li F. (2016). Structure, function, and evolution of coronavirus spike proteins. Annu. Rev. Virol..

[40] Xu C., Wang Y., Liu C., Zhang C., Han W., Hong X., Wang Y., Hong Q., Wang S., Zhao Q. (2021). Conformational dynamics of SARS-CoV-2 trimeric spike glycoprotein in complex with receptor ACE2 revealed by cryo-EM. Sci. Adv..

[41] Fallon L., Belfon K.A.A., Raguette L., Wang Y., Stepanenko D., Cuomo A., Guerra J., Budhan S., Varghese S., Corbo C.P. (2021). Free Energy Landscapes from SARS-CoV-2 Spike Glycoprotein Simulations Suggest that RBD Opening Can Be Modulated via Interactions in an Allosteric Pocket. J. Am. Chem. Soc..

[42] Wu Y., Qian R., Yang Y., Sheng Y., Li W., Wang W. (2021). Activation Pathways and Free Energy Landscapes of the SARS-CoV-2 Spike Protein. ACS Omega.

[43] Ray D., Le L., Andricioaei I. (2021). Distant residues modulate conformational opening in SARS-CoV-2 spike protein. Proc. Natl. Acad. Sci. USA.

[44] Govind Kumar V., Ogden D.S., Isu U.H., Polasa A., Losey J., Moradi M. (2022). Prefusion spike protein conformational changes are slower in SARS-CoV-2 than in SARS-CoV-1. J. Biol. Chem..

[45] Lu M., Uchil P.D., Li W., Zheng D., Terry D.S., Gorman J., Shi W., Zhang B., Zhou T., Ding S. (2020). Real-Time Conformational Dynamics of SARS-CoV-2 Spikes on Virus Particles. Cell Host Microbe.

[46] Sztain T., Ahn S.H., Bogetti A.T., Casalino L., Goldsmith J.A., Seitz E., Mccool R.S., Kearns F.L., Acosta-Reyes F., Maji S. (2021). A glycan gate controls opening of the SARS-CoV-2 spike protein. Nat. Chem..

[47] Bai C., Warshel A. (2020). Critical Differences between the Binding Features of the Spike Proteins of SARS-CoV-2 and SARS-CoV. J. Phys. Chem. B.

[48] Bai C., Wang J., Chen G., Zhang H., An K., Xu P., Du Y., Ye R.D., Saha A., Zhang A. (2021). Predicting Mutational Effects on Receptor Binding of the Spike Protein of SARS-CoV-2 Variants. J. Am. Chem. Soc..

[49] Walls A.C., Park Y.J., Tortorici M.A., Wall A., Mcguire A.T., Veesler D. (2020). Structure, function, and antigenicity of the SARS-CoV-2 spike glycoprotein. Cell.

[50] Watanabe Y., Allen J.D., Wrapp D., Mclellan J.S., Crispin M. (2020). Site-specific glycan analysis of the SARS-CoV-2 spike. Science.

[51] Mayer M.P., Schröder H., Rüdiger S., Paal K., Laufen T., Bukau B. (2000). Multistep mechanism of substrate binding determines chaperone activity of Hsp70. Nat. Struct. Biol..

[52] Callender R., Dyer R.B. (2015). The dynamical nature of enzymatic catalysis. Acc. Chem. Res..

[53] Granchi C. (2018). ATP citrate lyase (ACLY) inhibitors: An anti-cancer strategy at the crossroads of glucose and lipid metabolism. Eur. J. Med. Chem..

[54] Verschueren H.G., Blanchet C., Felix J., Dansercoer A., De Vos D., Bloch Y., Van Beeumen J., Svergun D., Gutsche I., Savvides S.N. (2019). Structure of ATP citrate lyase and the origin of citrate synthase in the Krebs cycle. Nature.

[55] Bazilevsky G.A., Affronti H.C., Wei X., Campbell S.L., Wellen K.E., Marmorstein R. (2019). ATP-citrate lyase multimerization is required for coenzyme-A substrate binding and catalysis. J. Biol. Chem..

[56] Wei J., Leit S., Kuai J., Therrien E., Rafi S., Harwood H.J., Delabarre B., Tong L. (2019). An allosteric mechanism for potent inhibition of human ATP-citrate lyase. Nature.

[57] Fan F., Williams H.J., Boyer J.G., Graham T.L., Zhao H., Lehr R., Qi H., Schwartz B., Raushel F.M., Meek T.D. (2012). On the catalytic mechanism of human ATP citrate lyase. Biochemistry.

[58] Mbaye M.N., Hou Q., Basu S., Teheux F., Pucci F., Rooman M. (2019). A comprehensive computational study of amino acid interactions in membrane proteins. Sci. Rep..

[59] Giliberti V., Badioli M., Nucara A., Calvani P., Ritter E., Puskar L., Aziz E.F., Hegemann P., Schade U., Ortolani M. (2017). Heterogeneity of the Transmembrane Protein Conformation in Purple Membranes Identified by Infrared Nanospectroscopy. Small.

[60] Timcenko M., Dieudonné T., Montigny C., Boesen T., Lyons J., Lenoir G., Nissen P. (2021). Structural Basis of Substrate-Independent Phosphorylation in a P4-ATPase Lipid Flippase. J. Mol. Biol..

[61] Bevers E.M., Williamson P.L. (2016). Getting to the Outer Leaflet: Physiology of Phosphatidylserine Exposure at the Plasma Membrane. Physiol. Rev..

[62] Kato U., Inadome H., Yamamoto M., Emoto K., Kobayashi T., Umeda M. (2013). Role for Phospholipid Flippase Complex of ATP8A1 and CDC50A Proteins in Cell Migration. J. Biol. Chem..

[63] Leventis P.A., Grinstein S. (2010). The Distribution and Function of Phosphatidylserine in Cellular Membranes. Annu. Rev. Biophys..

[64] Emoto K., Kobayashi T., Yamaji A., Aizawa H., Yahara I., Inoue K., Umeda M. (1996). Redistribution of phosphatidylethanolamine at the cleavage furrow of dividing cells during cytokinesis. Proc. Natl. Acad. Sci. USA.

[65] Zwaal R.F.A., Schroit A.J. (1997). Pathophysiologic Implications of Membrane Phospholipid Asymmetry in Blood Cells. Blood.

[66] Muthusamy B.P., Natarajan P., Zhou X., Graham T.R. (2009). Linking phospholipid flippases to vesicle-mediated protein transport. Biochim. Biophys. Acta.

[67] Eijnde S.M.V.D., Hoff M., Reutelingsperger C.P.M., Heerde W.L.V., Henfling M.E., Vermeij-Keers C., Schutte B., Borgers M., Ramaekers F.C. (2001). Transient expression of phosphatidylserine at cell-cell contact areas is required for myotube formation. J. Cell Sci..

[68] Baldridge R.D., Graham T.R. (2013). Two-gate mechanism for phospholipid selection and transport by type IV P-type ATPases. Proc. Natl. Acad. Sci. USA.

[69] Vestergaard A.L., Coleman J.A., Lemmin T., Mikkelsen S.A., Molday L.L., Vilsen B., Molday R.S., Dal Peraro M., Andersen J.P. (2014). Critical roles of isoleucine-364 and adjacent residues in a hydrophobic gate control of phospholipid transport by the mammalian P4-ATPase ATP8A2. Proc. Natl. Acad. Sci. USA.

[70] Bai L., You Q., Jain B.K., Duan H.D., Kovach A., Graham T.R., Li H. (2020). Transport mechanism of P4 ATPase phosphatidylcholine flippases. eLife.

[71] Hiraizumi M., Yamashita K., Nishizawa T., Nureki O. (2019). Cryo-EM structures capture the transport cycle of the P4-ATPase flippase. Science.

[72] Segawa K., Kurata S., Nagata S. (2018). The CDC50A extracellular domain is required for forming a functional complex with and chaperoning phospholipid flippases to the plasma membrane. J. Biol. Chem..

[73] Jensen M.S., Costa S.R., Duelli A.S., Andersen P.A., Poulsen L.R., Stanchev L.D., Gourdon P., Palmgren M., Gunther Pomorski T., Lopez-Marques R.L. (2017). Phospholipid flipping involves a central cavity in P4 ATPases. Sci. Rep..

[74] Hayashi S., Ueno H., Shaikh A.R., Umemura M., Kamiya M., Ito Y., Ikeguchi M., Komoriya Y., Iino R., Noji H. (2012). Molecular Mechanism of ATP Hydrolysis in F1-ATPase Revealed by Molecular Simulations and Single-Molecule Observations. J. Am. Chem. Soc..

[75] Zhou Y., Ojeda-May P., Nagaraju M., Pu J. (2016). Toward Determining ATPase Mechanism in ABC Transporters: Development of the Reaction Path-Force Matching QM/MM Method. Methods Enzymol..

[76] Van Der Velden L.M., Van De Graaf S.F.J., Klomp L.W.J. (2010). Biochemical and cellular functions of P4 ATPases. Biochem. J..

[77] Nakanishi H., Nishizawa T., Segawa K., Nureki O., Fujiyoshi Y., Nagata S., Abe K. (2020). Transport Cycle of Plasma Membrane Flippase ATP11C by Cryo-EM. Cell Rep..

[78] Kamerlin S.C.L., Vicatos S., Dryga A., Warshel A. (2011). Coarse-Grained (Multiscale) Simulations in Studies of Biophysical and Chemical Systems. Annu. Rev. Phys. Chem..

[79] Tang T., Bidon M., Jaimes J.A., Whittaker G.R., Daniel S. (2020). Coronavirus membrane fusion mechanism offers a potential target for antiviral development. Antiviral. Res..

[80] Warshel A., Weiss R.M. (1981). Empirical valence bond calculations of enzyme catalysis. Ann. N. Y. Acad. Sci..

[81] Sham Y.Y., Chu Z.T., Tao H., Warshel A. (2000). Examining methods for calculations of binding free energies: LRA, LIE, PDLD-LRA, and PDLD/S-LRA calculations of ligands binding to an HIV protease. Proteins.

